# A Novel Multidrug Resistant, Non-Tn*4401* Genetic Element-Bearing, Strain of *Klebsiella pneumoniae* Isolated From an Urban Lake With Drinking and Recreational Water Reuse

**DOI:** 10.3389/fmicb.2021.732324

**Published:** 2021-11-24

**Authors:** Luis Janssen, Felipe Marques de Almeida, Thais Amanda Silva Damasceno, Rodrigo de Paula Baptista, Georgios Joannis Pappas, Tatiana Amabile de Campos, Vicente de Paulo Martins

**Affiliations:** ^1^Department of Cellular Biology, Institute of Biological Sciences, University of Brasilia, Brasília, Brazil; ^2^Center for Tropical and Emerging Global Diseases, University of Georgia, Athens, GA, United States; ^3^Institute of Bioinformatics, University of Georgia, Athens, GA, United States

**Keywords:** *Klebsiella pneumoniae*, antimicrobial resistance, strain type, MLST, one health, water scarcity

## Abstract

Antimicrobial resistance (AMR) is an increasing and urgent issue for human health worldwide, as it leads to the reduction of available antibiotics to treat bacterial infections, in turn increasing hospital stays and lethality. Therefore, the study and genomic surveillance of bacterial carriers of resistance in and outside of clinical settings is of utter importance. A colony of multidrug resistant (MDR) bacteria identified as *Klebsiella* spp., by 16S rDNA amplicon sequencing, has been isolated from an urban lake in Brazil, during a drug-degrading bacterial prospection. Genomic analyses revealed the bacteria as *Klebsiella pneumoniae* species. Furthermore, the *in silico* Multilocus Sequence Typing (MLST) identified the genome as a new sequence type, ST5236. The search for antimicrobial resistance genes (ARGs) detected the presence of genes against beta-lactams, fosfomycin, acriflavine and efflux pumps, as well as genes for heavy metal resistance. Of particular note, an extended-spectrum beta-lactamase gene (*blaCTX-M-15*) has been detected in close proximity to *siphoviridae* genes, while a carbapenemase gene (*KPC-2*) has been found in an extrachromosomal contig, within a novel non-Tn4401 genetic element (NTE_KPC_). An extrachromosomal contig found in the V3 isolate is identical to a contig of a *K. pneumoniae* isolate from a nearby hospital, which indicates a putative gene flow from the hospital network into Paranoá lake. The discovery of a MDR isolate in this lake is worrisome, as the region has recently undergone periods of water scarcity causing the lake, which receives treated wastewater effluent, and is already used for recreational purposes, to be used as an environmental buffer for drinking water reuse. Altogether, our results indicate an underrepresentation of environmental *K. pneumoniae* among available genomes, which may hamper the understanding of the population dynamics of the species in the environment and its consequences in the spread of ARGs and virulence genes.

## Introduction

The World Health Organization (WHO) recognizes antimicrobial resistance (AMR) as an urgent global issue with impending increases in mortality rates, hospitalization length and cross-contamination risks, as well as overall economic losses (World Health Organization [WHO], 2015). In spite of this problem, global antibiotic gross consumption and consumption per capita increased 65 and 39%, respectively, from 2000 to 2015. The broad-spectrum penicillins presented the highest increase, followed by cephalosporins, quinolones, and macrolides (Klein et al., 2018). In the current scenario, that antimicrobials are present in hospitals, animal production and communities, as well as, disposed of as wastewater in sewers, water and soil, the WHO emphasizes the need of a multidisciplinary, “One Health” approach to tackle this issue (World Health Organization [WHO], 2015).

Fortunately, ever since the seminal work of John Snow, pointing to a public water pump as a source of a cholera outbreak (Snow, 1855), several technologies in microbiology have been developed, from bacterial isolation and culture to next generation sequencing platforms (NGS). As such, different methodologies, in particular the so-called genomic surveillance, have become important to track the dissemination of infectious microbes and their genes of relevance among humans, humans and animals and these two and the environment. NGS can be particularly useful in order to track the ever-increasing dissemination of antimicrobial resistance genes (ARGs) (Mitchell and Simner, 2019; Ransom et al., 2020).

Among the most common ARGs, there are the beta-lactamase genes. Two of the most important classes of beta-lactamases are the extended-spectrum beta-lactamases (ESBLs) and the carbapenemases. The CTX-M enzymes are members of the ESBLs, able to hydrolyze expanded-spectrum cephalosporins and monobactams (Cantón et al., 2012). Prevalent worldwide, the CTX-M beta-lactamases are the most common ESBLs, especially CTX-M-15 (Bevan et al., 2017; Bush and Bradford, 2020). Infections with ESBL producers often drive the prescription of carbapenems, which may promote the selection and spread of potentially untreatable carbapenemase-producing Enterobacterales (Bevan et al., 2017). Among carbapenemases, the KPC-2 and KPC-3 are the most widespread and most commonly reported genes (Stoesser et al., 2017; Zhang et al., 2020). These genes are frequently associated with mobile genetic elements (MGEs), such as plasmids and transposons. Thus far, the most common mobile element associated with *blaKPC*, at least for the species *Klebsiella pneumoniae*, is the transposon Tn*4401* (Yang et al., 2021). However, different transposable elements, broadly termed non-Tn4401 genetic elements (NTE_KPCs_) were first described by Shen et al. (2009). Since then, several NTE_KPCs_ sequences can be found deposited in GenBank, but the majority has not been formally described in articles (Yang et al., 2021). Thus, it becomes important for the study of *blaKPC* dispersal in hospitals and the community to describe them. There are reports of these elements in association with high-risk clonal group 258 lineages in Brazil (Cerdeira et al., 2019) as well as found in hospital-associated carbapenemase-resistant bacterial outbreaks in Colombia and Chile (Rada et al., 2020; Wozniak et al., 2020) and in wastewater (Gomi et al., 2018).

In an environmental perspective, Gillings et al. (2018) have coined the term xenogenetic DNA to represent novel gene arrangements, such as (MGEs) with ARGs, whose assembly and dispersion have been promoted by human activity, in analogy to xenobiotic chemical pollutants. In another analogy, xenogenetic DNA behave like invasive species in as much as their abundance is determined not only by release and transport, but also by replication. Thus, xenogenetic DNA could be understood as a novel type of pollutant, being able to replicate, but also being generated as a consequence of human activity.

*Klebsiella pneumoniae* is a gram-negative rod belonging to Enterobacterales. It is also part of the ESKAPEE group of pathogens (*Enterococcus faecium*, *Staphylococcus aureus*, *K. pneumoniae*, *Acinetobacter baumannii*, *Pseudomonas aeruginosa*, *Enterobacter* spp. and *Escherichia coli*) known for their capacity of causing nosocomial infections and ability to resist to multiple classes of antibiotics (Rice, 2008; Partridge et al., 2018). Moreover, some strains of *K. pneumoniae* are also known as hypervirulent, are community-acquired and capable to promote metastatic infections (Shon et al., 2013). Many of such strains belong to the Cluster groups (CGs) 258 and CG11. Of note, there are also strains whose phenotype has converged into acquiring both multidrug resistance and hypervirulence traits (Wyres et al., 2020).

Wyres and Holt (2018) have shown that *K. pneumoniae* have a tendency to harbor more AMR genes and plasmids than other ESKAPEE pathogens. Furthermore, the species has a broad ecological distribution, ranging from gut colonization in mammals to insects, plants, water bodies and soil. Lastly, clinically relevant lineages have been isolated from non-clinical contexts, such as domestic animals. Hence, the authors suggest that *K. pneumoniae* may serve as an important hub for acquisition and transmission, via horizontal gene transfer, of AMR genes to microbes in different environments. Because of this capacity of colonizing the animal gut and the external environment (water and soil) and of being frequent carriers of acquired resistance, Berendonk et al. (2015) propose *K. pneumoniae* as a primary bacterial indicator of AMR spread in the environment.

Taking the context of water bodies in Brazil, Oliveira et al. (2014) have isolated a KPC-2 producing *K. pneumoniae* belonging to the Sequence type (ST)11 clonal complex from rivers, which is frequently associated with hypervirulent strains. Dropa et al. (2016) have described a, at the time, new *K. pneumoniae* ST harboring CTX-M-8 beta-lactamase from a wastewater treatment plant (WWTP). Moreover, carbapenemase resistant *K. pneumoniae* have also been found in recreational waters in Rio de Janeiro (Montezzi et al., 2015; de Araujo et al., 2016; Paschoal et al., 2017) and carbapenemase resistant Enterobacteriales have been found in Santos Bay (Andrade et al., 2020).

In the present study we describe a multidrug resistant (MDR) NTC_KPC_-bearing *K. pneumoniae* strain belonging to a novel ST that has been found during a prospection for xenobiotic-degrading bacteria in an artificial urban lake, next to a WWTP.

## Materials and Methods

### Strain Isolation

A 50 mL sample was collected from the surface water of Paranoá lake (Brasília, Brazil: 15°44′27.4″S 47°52′52.8″W) and an aliquot of 50 μL was used as inoculum onto M9 minimum media containing acetaminophen as its only carbon source (90 mM Na_2_SO_4_, 22 mM KH_2_SO_4_, 18 mM NH_4_Cl, 2 mM MgSO_4_, 0.1 mM CaCl_2_, 15 g.L^–1^ agar, 3.3 mM acetaminophen). The resulting medium plate was incubated at 25°C for 24 h. For storage, the bacterial colony was inoculated in 5 mL of M9 liquid medium and incubated under agitation at 25°C and 180 rpm for 18 h. From this culture, 1.5 mL 25% (v/v) glycerol stocks were prepared and kept at −80°C. All samples were collected in accordance with Brazilian regulations and registered in the National System for the Management of Genetic Heritage and Associated Traditional Knowledge – SISGen (A373E15).

### Phenotypic Characterizations

An inoculum of 50 μL from the isolate culture, grown in LB medium at 25°C and 180 rpm for 18 h [10 g.L^–1^ triptone (Kasvi), 10 g.L^–1^ NaCl (Kasvi), 5 g.L^–1^ yeast extract (Kasvi)] was streaked onto MacConkey agar plates (Kasvi). Rugai medium was used for the presumptive biochemical identification, according to the manufacturer’s instructions (Laborclin).

The antibiotic sensitivity assay was performed using Müller-Hinton plates covered with different antibiotic-containing disks (Laborclin), including amikacin (30 μg), gentamicin (10 μg), tobramycin (10 μg), aztreonam (30 μg), cefepime (30 μg), ofloxacin (5 μg), norfloxacin (10 μg), ciprofloxacin (5 μg), levofloxacin (5 μg), lomefloxacin (10 μg), imipenem (10 μg), meropenem (10 μg), piperacillin/tazobactam (100/10 μg), and ticarcillin/clavulanic acid (85 μg). The antibiogram procedure was conducted and interpreted according to current EUCAST guidelines^1^, with *E. coli* ATCC 700336 as a negative control.

### Genus Identification by 16S Polymerase Chain Reaction *A*mplification and *S*equencing

The isolate was grown in 5 mL of LB media overnight at 37°C and 180 rpm. Genomic DNA was extracted using Wizard^®^ Genomic DNA purification kit (Promega), following the manufacturer’s instructions. Fragments of the 16S rRNA gene were amplified by polymerase chain reaction (PCR) from the genomic DNA using the primers RW01 (5′-AACTGGAGGAAGGTGGGGAT-3′) and DG74 (5′-AGGAGGTGATCCAACCGCA-3′), as described in Greisen et al. (1994). PCR reactions were performed in a 25 μL total volume incubated at 95°C for 4 min, 30 cycles of 95°C for 30 s, 58°C for 30 s, 72°C for 30 s and a final extension period of 72°C for 5 min. The resulting amplicons were subjected to Sanger sequencing in the High-Performance sequencing center at the Catholic University of Brasília.

### Next Generation Genome Sequencing

Genomic DNA was extracted following the same procedures for 16S amplification. Its integrity and purity were verified through 1% agarose gel electrophoresis and NanoDrop^TM^ Lite (Thermo Fisher Scientific) spectrophotometry. The DNA library was prepared using the rapid sequencing kit (RAD004) from Oxford Nanopore technologies (ONT) as per the manufacturer’s instructions and the sequencing reactions were performed in a R9.4.1 flowcell for 24 h, in a MinION device. Basecall was performed using Guppy 4.4.2.

### Genome Assembly and Annotation

*De novo* genome assembly was performed with Flye v2.8 (Kolmogorov et al., 2019) using the MpGAP pipeline v1.0^2^ using default parameters. Additionally, the assembled genome was polished (error correction step) with Medaka^3^ using the *r941_min_high_g344* model. After Medaka the genome was polished once more by Homopolish using the parameters for bacteria and R9.4 flowcell models (Huang et al., 2020). Genome completeness was assessed with BUSCO v4.1.0, using the enterobacterales_odb10 dataset (Seppey et al., 2019).

Genome annotation was performed with the bacannot pipeline v2.2^4^ using 85% gene coverage and identity thresholds for virulence gene (VG) annotations. With this pipeline, AMR genes were detected with CARD-RGI (database version 3.0.7) (Alcock et al., 2019), AMRFinderPlus v3.2.1 (Feldgarden et al., 2019) and ResFinder 4.0 (Bortolaia et al., 2020) tools, while VFDB (Liu et al., 2019) was used to identify virulence factors (accessed in July 2020). Prophage sequences were predicted with Phigaro v2.3.0 (Starikova et al., 2020). Capsule synthesis (K) and lipopolysaccharide (O) *loci* were further identified using Kaptive (Wick et al., 2018). Multilocus sequence typing (MLST) was performed with the *K. pneumoniae* MLST scheme (accessed in October 2020) (Diancourt et al., 2005). Plasmid replicons were detected with Plasmidfinder v2.1 (Carattoli et al., 2014) and Platon v1.5.0 (Schwengers et al., 2020).

### Comparative Genomics

Genome-based taxonomy analysis was performed with the Type Strain Genome Server (TYGS) (Meier-Kolthoff and Göker, 2019). FastANI (Jain et al., 2018) was used to calculate the average nucleotide identity (ANI) index against all the *K. pneumoniae* genomes from NCBI (Accessed in April 2021). Using all the genomes with at least 99 ANI score, a core genome phylogeny was reconstructed with Parsnp (Treangen et al., 2014). The resulting phylogenetic tree was visualized with ggtree (Oliveira et al., 2014) and re-rooted at midpoint for a better display. Moreover, we have added a clinical *K. pneumoniae* sample (Kp-BSB-A) that has been previously studied by our group (de Campos et al., 2018) to the dataset for comparative purposes. In order to standardize the results, the Kp-BSB-A genome was reannotated as KpBSB31 following the same methods as for KpV3. GrapeTree (Zhou et al., 2018) was used via the BIGSdb database^5^ to reconstruct and visualize the relationships among *K. pneumoniae* STs using the Minimum Spanning tree v2 (MSTreeV2) algorithm. Moreover, GCluster v2.0.6 (Li et al., 2020) was used to draw the figure with the gene cluster comparison of the *blaKPC* genes.

## Results

### Biological Characterizations

The sequencing of 16S fragment amplicons and biochemical assays of the isolate (KpV3) were compatible with *Klebsiella* spp. It also presented characteristic pink mucoid colonies in Macconkey agar medium. Antibiotic resistance profiles revealed that the bacterial isolate was only susceptible to aminoglycosides (amikacin, gentamicin, and tobramycin) (Supplementary Table 1). Therefore, it can be classified as a multidrug-resistant isolate (Magiorakos et al., 2012).

### Genomic Analyses

Sequence data obtained by long read nanopore DNA sequencing was used to assemble the genome of the studied strain. The resulting assembly comprised 5.4 Mb and showed great levels (98.5%) of gene space completeness based on BUSCO metrics (Table 1). The genome includes 5,132 CDS (coding sequences), 25 rRNA and 86 tRNA related sequences. Besides the chromosomal scaffold, two plasmid replicons, *ColRNAI* and *IncU*, have been detected. The genome was identified as *K. pneumoniae* by the TYGS, and this result was further validated with ANI analyses against available *Klebsiella* genomes in NCBI Refseq, showing the *K. pneumoniae* strain 2504 (GCF_011044895.1), from a study in Russia on hypervirulent *K. pneumoniae* isolates (PRJNA606163), as the closest genome available with 99.71 ANI index.

**TABLE 1:** KpV3 genome assembly statistics.

**Strain**	**Genome accession (NCBI)**	**Estimated long read Coverage**	**Assembly size (Mb)**	**Contigs (≥200 bp)**	**N50**	**BUSCO C; CS/CD/F/M**	**Plasmid replicons**
KpV3	GCA_019038575.1	475	5.4	7	5,363,194	98.5%; 98.0/0.5/0.7/0.8	ColRNAI, IncU

*The BUSCO Enterobacterales database (440 genes) was used to evaluate the completeness of the assembly. BUSCO numbers reported are percentage complete (C) followed by the percentages of complete single-copy (CS), complete duplicated (CD), fragmented (F), and missing (M) out of 440 genes.*

### Molecular Typing

Since some *Klebsiella* K and O serotypes are related to increased virulence particularly K1 and O1 serotypes, the identification of these *loci* is important for the rapid detection of high-risk clones (Paschoal et al., 2017; Andrade et al., 2020). Genome analysis enabled the classification of KpV3 strain as a KL45:O1v2 *K. pneumoniae* strain. The O1 serotype is one of the most common serotypes in clinically relevant *K. pneumoniae* isolates and is often associated with increased virulence (Hsieh et al., 2012; Fang et al., 2016), thus the identification of an O1 strain in an urban lake near the hospital is worrisome. Moreover, using the public BIGSdb *K. pneumoniae* MLST scheme the strain KpV3 was classified as a novel ST: 5236.

### Antimicrobial Resistance Genes and Virulence Genes

We performed the search for ARGs using three different tools and databases, namely AMRFinderPlus, CARD-RGI and Resfinder, in order to have a more comprehensive overview of the annotation, due to differences in database gene content and curation, as well as prediction schemes. All three software, in concert, detected genes for beta-lactams (*blaSHV-121*, *blaCTX-M-15*, and *blaKPC-2*) and fosfomycin (*fosA*) (Table 2). Moreover, several multidrug efflux pumps (*kdeA*, *emrD*, *oqxAB*, and *acrAB*) were also detected by at least one database. The efflux pump *oqxAB* has been regularly implicated in low to intermediate resistance to quinoxalines, quinolones, tigecycline, nitrofurantoin, several detergents and disinfectants (Li et al., 2019). Furthermore, the *acrAB* is an important intrinsic virulence factor, which have been shown to provide resistance to host-derived antimicrobial peptides in *E. coli* (Swick et al., 2011) and when overexpressed, contributes to multidrug resistance. Additionally, AMRFinderPlus also detected in the genome, genes conferring heavy metal resistance (*fieF* and *arsC*). Resfinder has identified multiple point mutations in the *acrR*, *ompK36*, and *ompK37* genes which are predicted to confer together resistance to fluoroquinolones, carbapenems and cephalosporins.

**TABLE 2:** Summary of KpV3 and KpBSB31 genes related to virulence and antimicrobial resistance.

**Characteristics**	**KpV3**	**KpBSB31**
Sequence type (ST)	5236	11
**Antimicrobial resistance to:**		
Beta-lactams	*blaCTX-M-15*, *blaSHV-121*, *blaKPC-2*	*blaSHV-182*, *blaKPC-2*, *blaLAP-2*
(Fluoro) quinolones	*oqxA*, *oqxB*	*oqxA*, *oqxB*, *qnrS1*
Fosfomycin	*fosA*	*fosA*
Sulfonamide		*sul1*, *sul2*
Tetracycline		*tetA*, *tetD*
Trimethoprim		*dfrA1*
Efflux pumps	*emrD*, *kdeA*, *acrA*, *acrB*	*emrD*, *kdeA*, *acrA*, *acrB*
**Heavy metal resistance genes to:**		
Iron stress	*fieF*	*fieF*
Arsenic	*arsC*	*arsC*
Quaternary ammonium compounds		*qacEdelta1*
Acid resistance		*asr*
**Virulence genes to:**		
Type 1 fimbriae	*fimABCDEFGHIK*	*fimABCDEFGHIK*
Type 3 fimbriae	*mrkABCDFHIJ*	*mrkABCDFHIJ*
Type VI secretion system	*tssBCDFGHIJKLM*, *tli1*, *tle1*	*tssABCDFGHIJKLM*, *tli1*, *tle1*
Enterobactin	*entABCEF*, *fepABCDG*, *fes*, *ybdA*	*entABCEF*, *fepABCDG*, *fes*, *ybdA*
Salmochelin	*iroE*	*iroE*
Yersiniabactin		*fyuA*, *irp1*, *ipr2*, *ybtAEPQSTUX*
Plasmids	*ColRNAI*, *IncU*	*Col440I*, *ColRNAI*, *IncA/C2*, *IncFIB(pKPHS1)*, *IncN*, *IncR*
Prophage-related sequences	*5*	*5*
GenBank accession number	*SAMN19730657*	*SAMEA104554881*

**FIGURE 1:**
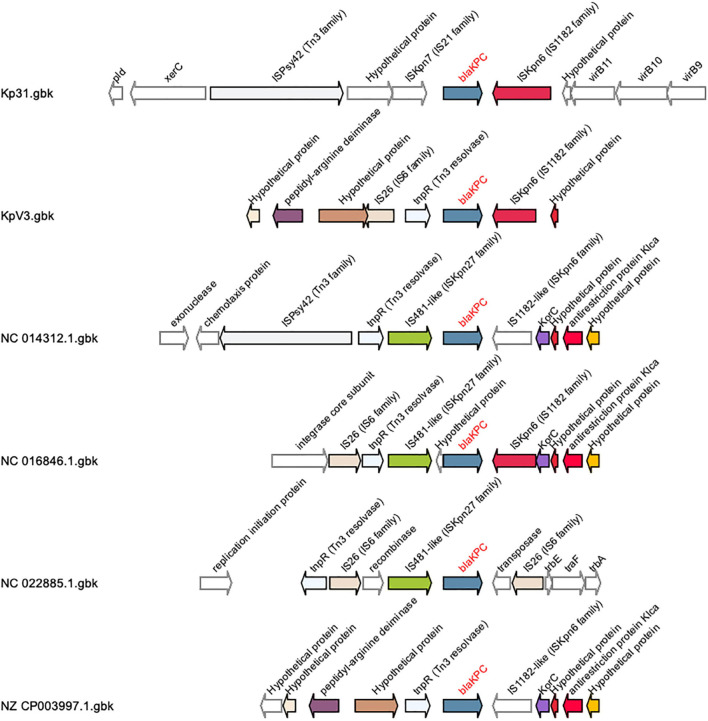
Schematic representation of group I NTE_KPCs_ gene clusters and its flanking genome contexts. This analysis was created with GCluster using different *Klebsiella pneumoniae* genomes, found in the literature, and the genomes of the strains KpV3 and Kp31. The *blaKPC* gene is represented in blue, the insertion sequences *ISkpn6* are colored in purple and it is located downstream the *blaKPC* gene, as in most NTE_KPCs_. The insertion sequence IS26 is represented in pink and is located upstream the *blaKPC*, but in the KpV3 this IS26 sequence is found in an opposite orientation when compared to the other group I NTE_KPCs_.

In terms of acquired resistance genes, a *bla-CTX-M-15* gene was found within the flanking region (3.3 kb distance) of a 39.2 kb *Siphoviridae* gene cluster in the bacterial chromosome, which could be a putative source to mobility to this gene. As originally discussed by Chen et al. (2014), the most common MGE associated with *blaKPC* in *K. pneumoniae* is the 4401 transposon (tn), while other MGEs were classified NTE_KPC_. Most NTE_KPCs_ share at least a truncated version of the *ISkpn6* insertion sequence gene downstream of *blaKPC*, with sub-type divisions depending on the gene composition found upstream of *blaKPC*, such as the presence of *blaTEM*. In KpV3, the *blaKPC-2* gene was found in association with a truncated insertion sequence (IS) ISKpn6, a Tn3 resolvase and an IS26, forming a putative NTE_KPC_ of approximately 3 kb (Figure 1). In this element, the IS 26 and *tnpR* resolvase are upstream of *blaKPC*, without *blaTEM* sequences, as it occurs with NTEKPC-Ib and NTEKPC-Id. However, the *ISkpn8* sequence is absent in the observed NTE_KPC_, which indicates that the element found in KpV3 is a putative novel group I NTE_KPC_. Furthermore, the IS26 is found in an opposite orientation when compared to the other NTE_KPC_ sequences, which could indicate an independent transposition event with this IS alone (Figure 1).

The search for VGs detected, in the chromosome of KpV3, the presence of VGs related to some classical *K. pneumoniae* virulence factors such as the phenolate siderophore enterobactin (*entABCEF*, *fepABCDG*, *fes*, and *ybdA*) and types I and III fimbriae (*fimABCDEFGHIK* and *mrkABCDFHIJ*) (Table 2). Moreover, the *iroE* (salmochelin siderophore) gene was also detected in the genome. The production of more than one type of siderophore is a characteristic of more virulent bacterium (Marr and Russo, 2019). Furthermore, several genes (*tssBCDFGHIJKLM*) related to the Type VI secretion system (T6SS) have also been detected. The T6SS is an apparatus related to bacterial competition, cell invasion and *in vivo* colonization, as well as DNA acquisition from other bacteria or metal acquisition from the environment, thus, capable of enhancing the bacterium environment fitness (Ho et al., 2014; Liu et al., 2017; Barbosa and Lery, 2019; Coulthurst, 2019). Upon visual inspection in Artemis genome browser and domain prediction from their putative proteins in CDvist (Adebali et al., 2015), we have found three VgrG4-like genes, which are T6SS effectors.

### Comparative Genomics

**FIGURE 2:**
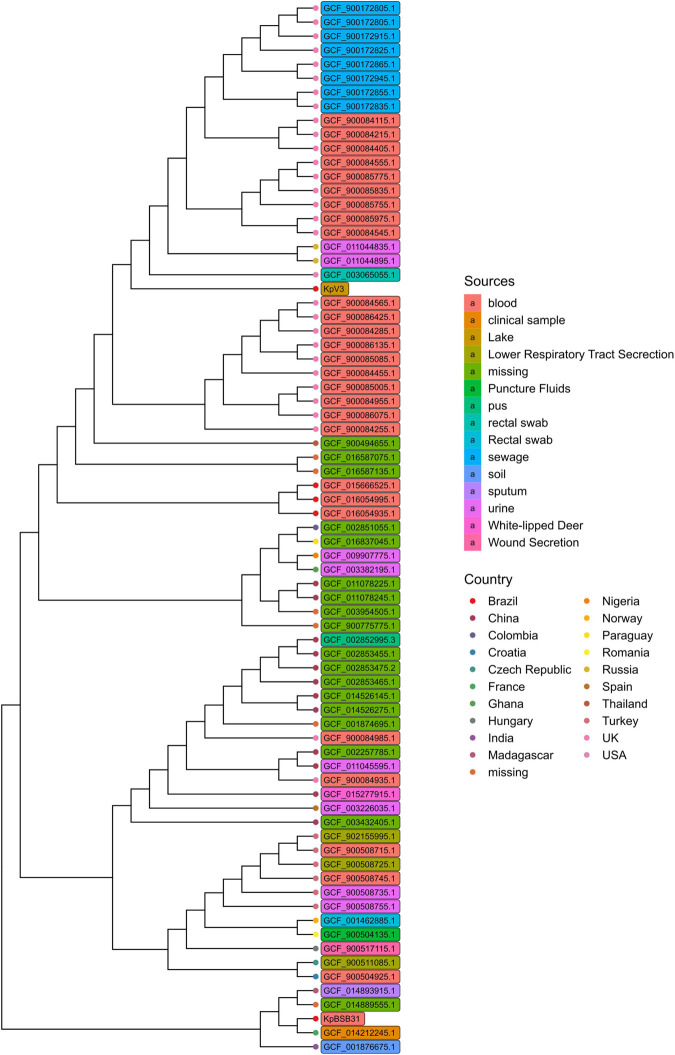
Core genome phylogeny reconstructed with Parsnp using genomes with at least 99 ANI score with KpV3. Branch tips and genome names have been respectively colored based on country and isolation sources.

A total of 74 *K. pneumoniae* genomes have been used to reconstruct a core genome phylogeny with Parsnp (Treangen et al., 2014; Figure 2). As expected, the KpV3 sample was placed as the single representative of a tree branch, meaning that closer genomes may exist but have not yet been identified. Most of the strains in the tree were isolated from clinical environments, which further indicates a bias toward sequencing of clinical isolates. Interestingly, although collected from very near locations, the KpV3 and KpBSB31 samples are placed distantly to each other, highlighting the genetic difference among clinical and environmental samples. In summary, these results emphasize the diversity of the *K. pneumoniae* species and underscore the need of more sequencing efforts on clinical and non-clinical samples to better understand the true extent of phylogenetic relationships in the species.

**FIGURE 3:**
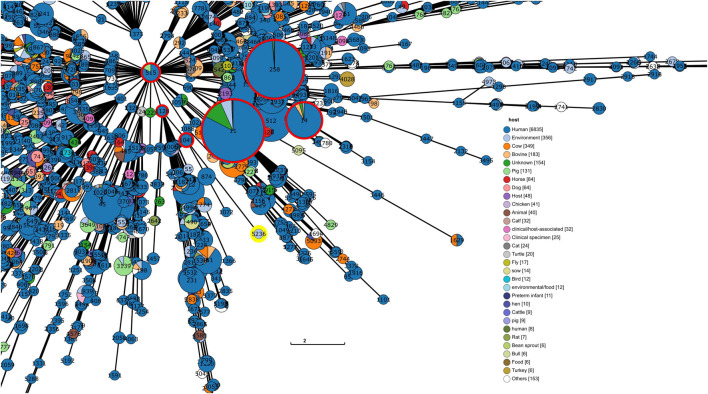
Section of the tree representation of genetic relationships among the different profiles of the *K. pneumoniae* MLST scheme. This analysis was produced via GrapeTree with the minimum spanning tree algorithm (MSTree V2). The ST identified in this study is highlighted with a yellow circle and high-risk lineages (CG 11, 14, and 258) are highlighted with red circles. For readability purposes, only the section of the tree where the ST is placed is shown. (The tree containing the whole analysis is in Supplementary Figure 1).

The reconstruction and visualization of relationships among *K. pneumoniae* STs enabled the observation that the ST 5236 (KpV3) does not have close relationships to worldwide threat STs such as ST 11, 14, and 258 (Figure 3). In fact, the results show the ST 5236 placed in a branch closer to STs 874, 1041, 1072, and 1128, in a bigger group of STs with 515 as founder. Among the closest STs, the majority of the deposited isolates comes from human hosts. This could indicate that either there is a bias toward isolation of hospital-born bacteria and/or that KpV3 could share important genomic features with hospital-born isolates.

For comparative purposes, we contrasted the presence/absence of ARGs and VGs with KpBSB31 (Table 2). The KpBSB31 (Kp-BSB-A) was isolated from the blood of a 60- to 70-year-old patient deceased 18 days after hospitalization (de Campos et al., 2018). The hospital from which the KpBSB31 sample was collected is very near to the KpV3 isolation site. Although we are analyzing and comparing only two isolates, the comparison between these samples could highlight the putative horizontal gene transfer between clinical and environmental samples. As expected, in terms of predicted gene content, the clinical sample KpBSB31 is more virulent and more resistant to antibiotics than the environmental sample KpV3, however it is possible to observe the presence and maintenance of relevant genes in KpV3 such as *iroE*, *blaCTX-M*, and *blaKPC*. Interestingly, the *ColRNAI* plasmid has been found to be almost identical (>99% identity) between samples. The maintenance of plasmids, VGs and ARGs in environmental strains is worrisome as they can act as a reservoir, playing a key role in the dissemination of different genetic traits (Huijbers et al., 2019; Fouz et al., 2020).

## Discussion

In the present study we have identified an environmental *K. pneumoniae* isolate belonging to a novel sequence type, ST 5236 and displaying capsular serotype (K) KL45 and LPS serotype (O) O1v2. With respect to the K antigens, there are currently 78 serotypes, despite having more than 130 allelic combinations in its biosynthetic *locus* (KLs) (Wick et al., 2018; Wyres et al., 2020). Among these serotypes, K1 and K2 are both most commonly isolated from patients, more virulent in mice experiments and more resistant to phagocytosis and intercellular killing by phagocytes (Paczosa and Mecsas, 2016). On the other hand, regarding the O antigens, there are nine serotypes and 12 O-*loci*, with serotypes O1 and O2 being the most common for clinical isolates (Wyres et al., 2020). Storey et al. (2020) have recently characterized a novel T6SS effector for *K. pneumoniae*, termed VgrG4. The presence of this gene in bacterial strains contributed to toxicity against bacterial and fungal species. Within this gene, the portion coding for the DUF2345 domain was responsible for the intoxication. In our study, we have found three genes with DUF2345 conserved regions, alongside a nearly complete T6SS gene cluster. The presence of multiple copies of DUF2345-bearing genes could represent a competitive edge for *K. pneumoniae* isolates directly against microbiotas from different environments or a strategy for quickly acquiring genes from them.

Although this notion has been long known by Brazilian indigenous people (Krenak, 2020), the “One World-One health” concept was established in 2004 as a form to understand human health. As this concept goes, human health is dependent on animal health, both domestic and wild, and on environmental health. Increasing, modern anthropic pressures on the environment, such as pollution, habitat destruction and others, promote changes in its composition, which ultimately leads to the increase in frequency and intensity of disease-states in humans (Destoumieux-Garzón et al., 2018).

Nevertheless, the sequencing efforts for *K. pneumoniae* are mainly focused on clinical samples, a bias reflected in the phylogenetic analysis presented in Figure 3 where KpV3 has been placed in an undivided branch and the majority of bacterial isolates in the tree come from human samples. Besides, the samples derived from the sewer were actually taken from hospital effluents. Not only the AMR gene profile found in WWTPs reflects that of clinical settings (Pärnänen et al., 2019), but surveillance in WWTPs could represent a more accurate perspective of AMR spread at a populational level (Sims and Kasprzyk-Hordern, 2020). Moreover, a similar pattern was also observed in the MLST tree analysis (Figure 3) where the branch length represents the distance between groups. The analysis shows that the ST 5236 (KpV3) has a reasonable distance to its closest ST (ST 1041), which means that closer STs might exist, but are not yet identified. It is important to observe that this new ST is not related to other worldwide threat STs such as ST 11 and 258, meaning that this new ST might not be a threat in terms of hypervirulence.

It is disturbing that we have found an environmental *K. pneumoniae* strain containing both an ESBL (CTX-M-15) and a carbapenemase (KPC-2) enzyme, which raises concern about the selective pressure applied in the region. Of special note, both genes have been found inside or very near MGEs. The *bla-CTX-M-15* was found in the bacterial chromosome, close to a putative *Siphoviridae* prophage sequence. This viral family has been associated with human fecal pollution in water bodies and with beta-lactamase gene transfer (Colomer-Lluch et al., 2011). Furthermore, the *blaKPC2* gene was found in association with a transposase gene, forming a small gene cluster with two other genes. *Klebsiella* spp. ISs are often associated with AMR genes (Razavi et al., 2020) and transposition events can be an important factor in spreading these genes in contexts of positive selection among different plasmids, strains and species (Sheppard et al., 2016).

When comparing the genomes of the environmental KpV3 and the clinical isolate KpBSB31, we have detected that the *ColRNAI* plasmid is identical between the samples, which indicates a putative genetic flow of mobile elements between clinical and environmental isolates in the lake near the hospital. Similar observations of gene flows from hospitals water bodies have been made elsewhere (Ekwanzala et al., 2019; Lepuschitz et al., 2019; Bleichenbacher et al., 2020). However, it is unclear whether KpV3 isolate represents a clonal dispersion of hospital *K. pneumoniae* or if it has acquired genetic features from hospital-borne isolates via HGT. The extent of this genetic flow should be addressed by sampling different parts of the hospital system wastewater treatment. Thorough analyses of microbes discharged from the WWTPs into Paranoá lake are required to access the extent of their contribution to dissemination of AMR. Furthermore, the quantification of antibiotics and heavy metals in WWTPs, which may select/co-select resistant microbes (Hernando-Amado et al., 2019), is also of relevance, though fecal pollution alone may be the main factor in AMR spread (Karkman et al., 2019). Finally, Ekwanzala et al. (2019) have shown that in a river which receives WWTP effluent, a larger ratio of carbapenem resistant versus susceptible *K. pneumoniae* strains was found in its sediment, in contrast to surface waters. Thus, different portions of a given water body could weigh more in AMR maintenance. Danko et al. (2021) have analyzed >4,000 metagenomic samples from 60 cities around the world, as to characterize their urban microbiomes. Even with these many samples, the rarefaction curves for microbial species and ARGs did not saturate, indicating there is a considerable amount of diversity to be explored, particularly when considering the selective pressures for the formation of new ARGs and MGEs.

Brasília is located in a morphoclimatic domain, termed Cerrado, with two well-defined seasons, wet and dry. As a consequence of the dry season, the city has undergone into situations of water scarcity in recent years. As a response, Paranoá lake, which receives treated effluent from two WWTPs, has been turned into an environmental buffer for water reuse, including drinking water (Sodré and Sampaio, 2020). Hence, the discovery of an ESBL producer carbapenem-resistant *K. pneumoniae* isolate in this lake is of great concern, as regions undergoing water scarcity might become more susceptible to AMR spread, despite the presence of downstream water treatment processes. Additionally, climate change models based upon Intergovernmental Panel on Climate Change (IPCC) scenarios predict increases in temperature and dry season duration as well as precipitation decreases for the Cerrado biome (Bustamante et al., 2012). MacFadden et al. (2018) have shown that increases in minimal local temperatures are associated with higher frequency of infections caused by antimicrobial-resistant *E. coli*, *S. aureus*, and *K. pneumoniae*. Also, Anderson et al. (2008) have shown that *K. pneumoniae* blood stream infections occur more commonly with higher temperatures and dew point. There are many, non-excluding hypotheses for the causes of this correlation, from bacterial physiology to human societal changes (Rodríguez-Verdugo et al., 2020). Collignon et al. (2018) point that socioeconomical factors such poor infrastructure and governance, low health expenditure and high GDP and education were associated with higher AMR levels around the world. As the authors discuss, while temperature was positively correlated with AMR, this could be a correlation by proxy (Such as poor infrastructure) or a direct correlation. As water reuse may become more common as a result of climate change (Tram et al., 2014), this phenomenon may play a key role in AMR spread and warrants further inquiry.

Altogether, we have little evidence in favor of KpV3 as an isolate with high virulence. However, our observations suggest that there is a gene flow from hospitals into the lake, likely through WWTPs. While our findings point toward this hypothesis, it has been based so far on a single isolate. Therefore, the extent of ARG dissemination into the lake and how it may be represented in other isolates are a matter of future studies. Furthermore, there may be a positive selective pressure being applied in the Paranoá lake that may promote the selection and spread of potentially untreatable carbapenemase-producing bacteria while turning the region as a possible reservoir for ARGs. Thus, genomic surveillance and quality assessment programs with the wastewaters that are dumped in the lake are required to control and mitigate such pressure.

## Data Availability Statement

The dataset whole genome sequencing data for the KpV3 isolate is available under the NCBI Bioproject PRJNA738490, and for the Kp-BSB-A (KpBSB31) is available under the NCBI Bioproject PRJEB24576 (de Campos et al., 2018).

## Author Contributions

LJ and TD performed the strain isolation and phenotypic characterizations. LJ, FA, and RB performed the genome sequencing and bioinformatics analyses. GP, TC, and VM conceived and supervised the study. LJ, FA, GP, TC, and VM wrote the manuscript and analyzed the data. All authors contributed to the article and approved the submitted version.

## Conflict of Interest

The authors declare that the research was conducted in the absence of any commercial or financial relationships that could be construed as a potential conflict of interest.

## Publisher’s Note

All claims expressed in this article are solely those of the authors and do not necessarily represent those of their affiliated organizations, or those of the publisher, the editors and the reviewers. Any product that may be evaluated in this article, or claim that may be made by its manufacturer, is not guaranteed or endorsed by the publisher.

## Footnotes

1
https://eucast.org/

2
https://github.com/fmalmeida/MpGAP

3
https://github.com/nanoporetech/medaka

4
https://github.com/fmalmeida/bacannot

5
http://bigsdb.web.pasteur.fr/klebsiella/klebsiella.html

## Supplementary Material

Supplementary Figure 1 Tree representation of genetic relationships among the different profiles of the *K. pneumoniae* MLST scheme. This analysis was produced via GrapeTree with the minimum spanning tree algorithm (MSTree V2). The ST identified in this study is highlighted with a yellow circle and a red arrow. High-risk lineages (CG 11, 14, and 258) are highlighted with red circles.

## References

[B1] Adebali O. Ortega D. R. Zhulin I. B. (2015). CDvist: a webserver for identification and visualization of conserved domains in protein sequences. *Bioinformatics* 31 1475–1477. 10.1093/bioinformatics/btu836 25527097 PMC4410658

[B2] Alcock B. P. Raphenya A. R. Lau T. T. Y. Tsang K. K. Bouchard M. Edalatmand A. (2019). CARD 2020: antibiotic resistome surveillance with the comprehensive antibiotic resistance database. *Nucleic Acids Res.* 48 D517–D525. 10.1093/nar/gkz935 PMC7145624 31665441

[B3] Anderson D. J. Richet H. Chen L. F. Spelman D. W. Hung Y. Huang A. T. (2008). Seasonal variation in *Klebsiella pneumoniae* bloodstream infection on 4 continents. *J. Infect. Dis.* 197 752–756. 10.1086/527486 18260762

[B4] Andrade V. C. Caetano T. Mendo S. Oliveira A. J. F. C. (2020). Carbapenem resistant *Enterobacteriaceae* from port areas in São Paulo State (Brazil): isolation and molecular characterization. *Mar. Pollut. Bull.* 159:111329. 10.1016/j.marpolbul.2020.111329 32777543

[B5] Barbosa V. A. A. Lery L. M. S. (2019). Insights into *Klebsiella pneumoniae* type VI secretion system transcriptional regulation. *BMC Genomics.* 20:506. 10.1186/s12864-019-5885-9 31215404 PMC6580597

[B6] Berendonk T. U. Manaia C. M. Merlin C. Fatta-Kassinos D. Cytryn E. Walsh F. (2015). Tackling antibiotic resistance: the environmental framework. *Nat. Rev. Microbiol.* 13 310–317. 25817583 10.1038/nrmicro3439

[B7] Bevan E. R. Jones A. M. Hawkey P. M. (2017). Global epidemiology of CTX-M β-lactamases: temporal and geographical shifts in genotype. *J. Antimicrob. Chemother.* 72 2145–2155. 10.1093/jac/dkx146 28541467

[B8] Bleichenbacher S. Stevens M. J. A. Zurfluh K. Perreten V. Endimiani A. Stephan R. (2020). Environmental dissemination of carbapenemase-producing *Enterobacteriaceae* in rivers in Switzerland. *Environ. Pollut.* 265:115081. 10.1016/j.envpol.2020.115081 32806462

[B9] Bortolaia V. Kaas R. S. Ruppe E. Roberts M. C. Schwarz S. Cattoir V. (2020). ResFinder 4.0 for predictions of phenotypes from genotypes. *J. Antimicrob. Chemother.* 75 3491–3500. 10.1093/jac/dkaa345 32780112 PMC7662176

[B10] Bush K. Bradford P. A. (2020). Epidemiology of β-Lactamase-Producing pathogens. *Clin. Microbiol. Rev.* 33 e47–e19. 10.1128/CMR.00047-19 PMC7048014 32102899

[B11] Bustamante M. Nardoto G. Pinto A. Resende J. Takahashi F. Vieira L. (2012). Potential impacts of climate change on biogeochemical functioning of Cerrado ecosystems. *Braz. J. Biol.* 72(Suppl. 3) 655–671. 10.1590/s1519-69842012000400005 23011296

[B12] Cantón R. González-Alba J. M. Galán J. C. (2012). CTX-M enzymes: origin and diffusion. *Front. Microbiol.* 3:110. 10.3389/fmicb.2012.00110 22485109 PMC3316993

[B13] Carattoli A. Zankari E. García-Fernández A. Voldby Larsen M. Lund O. Villa L. (2014). *In Silico* detection and typing of plasmids using plasmidfinder and plasmid multilocus sequence typing. *Antimicrob. Agents Chemother.* 58 3895–3903. 10.1128/AAC.02412-14 24777092 PMC4068535

[B14] Cerdeira L. T. Lam M. M. C. Wyres K. L. Wick R. R. Judd L. M. Lopes R. (2019). Small IncQ1 and Col-like plasmids harboring *bla* _*KPC–2*_ and Non-Tn *4401* elements (NTE _*KPC*_ -IId) in high-risk lineages of *Klebsiella pneumoniae* CG258. *Antimicrob. Agents Chemother.* 63 1–4. 10.1128/AAC.02140-18 30602517 PMC6395902

[B15] Chen L. Mathema B. Chavda K. D. DeLeo F. R. Bonomo R. A. Kreiswirth B. N. (2014). Carbapenemase-producing *Klebsiella pneumoniae*: molecular and genetic decoding. *Trends Microbiol.* 22 686–696. 10.1016/j.tim.2014.09.003 25304194 PMC4365952

[B16] Collignon P. Beggs J. J. Walsh T. R. Gandra S. Laxminarayan R. (2018). Anthropological and socioeconomic factors contributing to global antimicrobial resistance: a univariate and multivariable analysis. *Lancet Planetary Health.* 2 e398–e405. 10.1016/S2542-5196(18)30186-4 30177008

[B17] Colomer-Lluch M. Jofre J. Muniesa M. (2011). Antibiotic resistance genes in the bacteriophage DNA fraction of environmental samples. Aziz R, organizador. *PLoS One* 6:e17549. 10.1371/journal.pone.0017549 21390233 PMC3048399

[B18] Coulthurst S. (2019). The Type VI secretion system: a versatile bacterial weapon. *Microbiology* 165 503–515. 10.1099/mic.0.000789 30893029

[B19] Danko D. Bezdan D. Afshin E. E. Ahsanuddin S. Bhattacharya C. Butler D. J. (2021). A global metagenomic map of urban microbiomes and antimicrobial resistance. *Cell* 184 3376.e17–3393.e17. 10.1016/j.cell.2021.05.002 34043940 PMC8238498

[B20] de Araujo C. F. M. Silva D. M. Carneiro M. T. Ribeiro S. Fontana-Maurell M. Alvarez P. (2016). Detection of carbapenemase genes in aquatic environments in Rio de Janeiro, Brazil. *Antimicrob. Agents Chemother.* 60 4380–4383. 10.1128/AAC.02753-15 27139469 PMC4914687

[B21] de Campos T. A. Gonçalves L. F. Magalhães K. G. de Paulo Martins V. Pappas Júnior G. J. Peirano G. (2018). A fatal bacteremia caused by hypermucousviscous KPC-2 producing extensively drug-resistant K64-ST11 *Klebsiella pneumoniae* in Brazil. *Front Med.* 5:265. 10.3389/fmed.2018.00265 30298131 PMC6161680

[B22] Destoumieux-Garzón D. Mavingui P. Boetsch G. Boissier J. Darriet F. Duboz P. (2018). The one health concept: 10 years old and a long road ahead. *Front. Vet. Sci.* 5:14. 10.3389/fvets.2018.00014 29484301 PMC5816263

[B23] Diancourt L. Passet V. Verhoef J. Grimont P. A. D. Brisse S. (2005). Multilocus sequence typing of *Klebsiella pneumoniae* nosocomial isolates. *J. Clin. Microbiol.* 43 4178–4182. 10.1128/JCM.43.8.4178-4182.2005 16081970 PMC1233940

[B24] Dropa M. Lincopan N. Balsalobre L. C. Oliveira D. E. Moura R. A. Fernandes M. R. (2016). Genetic background of novel sequence types of CTX-M-8- and CTX-M-15-producing *Escherichia coli* and *Klebsiella pneumoniae* from public wastewater treatment plants in São Paulo, Brazil. *Environ. Sci. Pollut. Res.* 23 4953–4958. 10.1007/s11356-016-6079-5 26782324

[B25] Ekwanzala M. D. Dewar J. B. Kamika I. Momba M. N. B. (2019). Tracking the environmental dissemination of carbapenem-resistant *Klebsiella pneumoniae* using whole genome sequencing. *Sci. Tot. Environ.* 691 80–92. 10.1016/j.scitotenv.2019.06.533 31319261

[B26] Fang C.-T. Shih Y.-J. Cheong C.-M. Yi W.-C. (2016). Rapid and accurate determination of lipopolysaccharide O-antigen types in *Klebsiella pneumoniae* with a Novel PCR-based O-genotyping method. Munson E, organizador. *J. Clin. Microbiol.* 54 666–675. 10.1128/JCM.02494-15 26719438 PMC4767969

[B27] Feldgarden M. Brover V. Haft D. H. Prasad A. B. Slotta D. J. Tolstoy I. (2019). Validating the AMRFinder tool and resistance gene database by using antimicrobial resistance genotype-phenotype correlations in a collection of isolates. *Antimicrob. Agents Chemother.* 63 e483–e419. 10.1128/AAC.00483-19 PMC6811410 31427293

[B28] Fouz N. Pangesti K. N. A. Yasir M. Al-Malki A. L. Azhar E. I. Hill-Cawthorne G. A. (2020). The contribution of wastewater to the transmission of antimicrobial resistance in the environment: implications of mass gathering settings. *Trop. Med. Infect. Dis.* 5:33. 10.3390/tropicalmed5010033 32106595 PMC7157536

[B29] Gillings M. R. Westoby M. Ghaly T. M. (2018). Pollutants that replicate: xenogenetic DNAs. *Trends Microbiol.* 26 975–977. 10.1016/j.tim.2018.08.003 30170783

[B30] Gomi R. Matsuda T. Yamamoto M. Chou P.-H. Tanaka M. Ichiyama S. (2018). Characteristics of carbapenemase-producing *Enterobacteriaceae* in wastewater revealed by genomic analysis. *Antimicrob. Agents Chemother.* 62 e2501–e2517. 10.1128/AAC.02501-17 PMC5923170 29483120

[B31] Greisen K. Loeffelholz M. Purohit A. Leong D. (1994). PCR primers and probes for the 16S rRNA gene of most species of pathogenic bacteria, including bacteria found in cerebrospinal fluid. *J. Clin. Microbiol.* 32 335–351. 10.1128/jcm.32.2.335-351.1994 7512093 PMC263034

[B32] Hernando-Amado S. Coque T. M. Baquero F. Martínez J. L. (2019). Defining and combating antibiotic resistance from One Health and Global Health perspectives. *Nat. Microbiol.* 4 1432–1442. 10.1038/s41564-019-0503-9 31439928

[B33] Ho B. T. Dong T. G. Mekalanos J. J. (2014). A view to a kill: the bacterial type VI secretion system. *Cell Host Microbe* 15 9–21. 10.1016/j.chom.2013.11.008 24332978 PMC3936019

[B34] Hsieh P.-F. Lin T.-L. Yang F.-L. Wu M.-C. Pan Y.-J. Wu S.-H. (2012). Lipopolysaccharide O1 antigen contributes to the virulence in *Klebsiella pneumoniae* causing pyogenic liver abscess. Forestier C, organizador. *PLoS One.* 7:e33155. 10.1371/journal.pone.0033155 22427976 PMC3299736

[B35] Huang Y.-T. Liu P.-Y. Shih P.-W. (2020). Homopolish: a method for the removal of systematic errors in nanopore sequencing by homologous polishing. *Genome Biol.* 22:95. 10.1186/s13059-021-02282-6 33789731 PMC8011154

[B36] Huijbers P. M. C. Flach C.-F. Larsson D. G. J. (2019). A conceptual framework for the environmental surveillance of antibiotics and antibiotic resistance. *Environ. Int.* 130:104880. 10.1016/j.envint.2019.05.074 31220750

[B37] Jain C. Rodriguez-R L. M. Phillippy A. M. Konstantinidis K. T. Aluru S. (2018). High throughput ANI analysis of 90K prokaryotic genomes reveals clear species boundaries. *Nat. Commun.* 9:5114. 30504855 10.1038/s41467-018-07641-9 PMC6269478

[B38] Karkman A. Pärnänen K. Larsson D. G. J. (2019). Fecal pollution can explain antibiotic resistance gene abundances in anthropogenically impacted environments. *Nat. Commun.* 10:80. 10.1038/s41467-018-07992-3 30622259 PMC6325112

[B39] Klein E. Y. Van Boeckel T. P. Martinez E. M. Pant S. Gandra S. Levin S. A. (2018). Global increase and geographic convergence in antibiotic consumption between 2000 and 2015. *Proc. Natl. Acad. Sci. U.S.A.* 115 E3463–E3470. 10.1073/pnas.1717295115 29581252 PMC5899442

[B40] Kolmogorov M. Yuan J. Lin Y. Pevzner P. A. (2019). Assembly of long, error-prone reads using repeat graphs. *Nat. Biotechnol.* 37:12. 30936562 10.1038/s41587-019-0072-8

[B41] Krenak A. (2020). *Ideas to Postpone the End of the World.* Toronto: House of Anansi Press, 88.

[B42] Lepuschitz S. Schill S. Stoeger A. Pekard-Amenitsch S. Huhulescu S. Inreiter N. (2019). Whole genome sequencing reveals resemblance between ESBL-producing and carbapenem resistant *Klebsiella pneumoniae* isolates from Austrian rivers and clinical isolates from hospitals. *Sci. Tot. Environ.* 662 227–235. 10.1016/j.scitotenv.2019.01.179 30690357

[B43] Li J. Zhang H. Ning J. Sajid A. Cheng G. Yuan Z. (2019). The nature and epidemiology of OqxAB, a multidrug efflux pump. *Antimicrob. Resist. Infect. Control.* 8:44. 30834112 10.1186/s13756-019-0489-3 PMC6387526

[B44] Li X. Chen F. Chen Y. (2020). Gcluster: a simple-to-use tool for visualizing and comparing genome contexts for numerous genomes. Robinson P, organizador. *Bioinformatics.* 36 3871–3873. 10.1093/bioinformatics/btaa212 32221617

[B45] Liu B. Zheng D. Jin Q. Chen L. Yang J. (2019). VFDB 2019: a comparative pathogenomic platform with an interactive web interface. *Nucleic Acids Res.* 47 D687–D692. 10.1093/nar/gky1080 30395255 PMC6324032

[B46] Liu L. Ye M. Li X. Li J. Deng Z. Yao Y.-F. (2017). Identification and characterization of an antibacterial Type VI secretion system in the carbapenem-resistant strain *Klebsiella pneumoniae* HS11286. *Front. Cell Infect. Microbiol.* 7:442. 10.3389/fcimb.2017.00442 29085808 PMC5649205

[B47] MacFadden D. R. McGough S. Fisman D. Santillana M. Brownstein J. (2018). Antibiotic resistance increases with local temperature. *Nat. Clim. Change* 8:6. 10.1038/s41558-018-0161-6 PMC6201249 30369964

[B48] Magiorakos A.-P. Srinivasan A. Carey R. B. Carmeli Y. Falagas M. E. Giske C. G. (2012). Multidrug-resistant, extensively drug-resistant and pandrug-resistant bacteria: an international expert proposal for interim standard definitions for acquired resistance. *Clin. Microbiol. Infect.* 18 268–281. 10.1111/j.1469-0691.2011.03570.x 21793988

[B49] Marr C. M. Russo T. A. (2019). Hypervirulent *Klebsiella pneumoniae*: a new public health threat. *Expert Rev. Anti-Infective Ther.* 17 71–73. 10.1080/14787210.2019.1555470 30501374 PMC6349525

[B50] Meier-Kolthoff J. P. Göker M. (2019). TYGS is an automated high-throughput platform for state-of-the-art genome-based taxonomy. *Nat. Commun.* 10:2182. 10.1038/s41467-019-10210-3 31097708 PMC6522516

[B51] Mitchell S. L. Simner P. J. (2019). Next-Generation sequencing in clinical microbiology. *Clin. Lab. Med.* 39 405–418. 31383265 10.1016/j.cll.2019.05.003

[B52] Montezzi L. F. Campana E. H. Corrêa L. L. Justo L. H. Paschoal R. P. da Silva I. L. V. D. (2015). Occurrence of carbapenemase-producing bacteria in coastal recreational waters. *Int. J. Antimicrob. Agents* 45 174–177. 10.1016/j.ijantimicag.2014.10.016 25499185

[B53] Oliveira S. Moura R. Silva K. Pavez M. McCulloch J. Dropa M. (2014). Isolation of KPC-2-producing *Klebsiella pneumoniae* strains belonging to the high-risk multiresistant clonal complex 11 (ST437 and ST340) in urban rivers. *J. Antimicrob. Chemother.* 69 849–851. 10.1093/jac/dkt431 24159156

[B54] Paczosa M. K. Mecsas J. (2016). *Klebsiella pneumoniae*: going on the offense with a strong defense. *Microb. Mol. Biol. Rev.* 80 629–661. 10.1128/MMBR.00078-15 27307579 PMC4981674

[B55] Pärnänen K. M. M. Narciso-da-Rocha C. Kneis D. Berendonk T. U. Cacace D. Do T. T. (2019). Antibiotic resistance in European wastewater treatment plants mirrors the pattern of clinical antibiotic resistance prevalence. *Sci. Adv.* 5:eaau9124. 10.1126/sciadv.aau9124 30944853 PMC6436925

[B56] Partridge S. R. Kwong S. M. Firth N. Jensen S. O. (2018). Mobile genetic elements associated with antimicrobial resistance. *Clin. Microbiol. Rev.* 31 e88–e17. 10.1128/CMR.00088-17 PMC6148190 30068738

[B57] Paschoal R. P. Campana E. H. Corrêa L. L. Montezzi L. F. Barrueto L. R. L. da Silva I. R. (2017). Concentration and variety of carbapenemase producers in recreational coastal waters showing distinct levels of pollution. *Antimicrob. Agents Chemother.* 61 e1963–e1917. 10.1128/AAC.01963-17 28971868 PMC5700321

[B58] Rada A. M. De La Cadena E. Agudelo C. Capataz C. Orozco N. Pallares C. (2020). Dynamics of *bla* _*KPC–2*_ dissemination from Non-CG258 *Klebsiella pneumoniae* to other *Enterobacterales* via IncN plasmids in an area of high endemicity. *Antimicrob. Agents Chemother.* 64 e1743–e1720. 10.1128/AAC.01743-20 32958711 PMC7674068

[B59] Ransom E. M. Potter R. F. Dantas G. Burnham C.-A. D. (2020). Genomic Prediction of antimicrobial resistance: ready or not, here it comes! *Clin. Chem.* 66 1278–1289. 10.1093/clinchem/hvaa172 32918462

[B60] Razavi M. Kristiansson E. Flach C.-F. Larsson D. G. J. (2020). The association between insertion sequences and antibiotic resistance genes. *mSphere* 5 e418–e420. 10.1128/mSphere.00418-20 PMC7471000 32878926

[B61] Rice L. B. (2008). Federal funding for the study of antimicrobial resistance in nosocomial pathogens: no eskape. *J. Infect. Dis.* 197 1079–1081. 10.1086/533452 18419525

[B62] Rodríguez-Verdugo A. Lozano-Huntelman N. Cruz-Loya M. Savage V. Yeh P. (2020). Compounding effects of climate warming and antibiotic resistance. *iScience* 23:101024. 10.1016/j.isci.2020.101024 32299057 PMC7160571

[B63] Schwengers O. Barth P. Falgenhauer L. Hain T. Chakraborty T. Goesmann A. (2020). Platon: identification and characterization of bacterial plasmid contigs in short-read draft assemblies exploiting protein sequence-based replicon distribution scores. *Microb. Genom.* 1–12. 10.1099/mgen.0.000398 32579097 PMC7660248

[B64] Seppey M. Manni M. Zdobnov E. M. (2019). “BUSCO: assessing genome assembly and annotation completeness,” in *Gene Prediction (Methods in Molecular Biology*, Vol. 1962 ed. Kollmar M. (New York, NY: Springer), 227–245. 10.1007/978-1-4939-9173-0_14 31020564

[B65] Shen P. Wei Z. Jiang Y. Du X. Ji S. Yu Y. (2009). Novel genetic environment of the carbapenem-hydrolyzing β-Lactamase KPC-2 among *Enterobacteriaceae* in China. *Antimicrob. Agents Chemother.* 53 4333–4338. 10.1128/AAC.00260-09 19620332 PMC2764158

[B66] Sheppard A. E. Stoesser N. Wilson D. J. Sebra R. Kasarskis A. Anson L. W. (2016). Nested russian doll-like genetic mobility drives rapid dissemination of the carbapenem resistance gene *bla* _*KPC*_. *Antimicrob. Agents Chemother.* 60 3767–3778. 10.1128/AAC.00464-16 27067320 PMC4879409

[B67] Shon A. S. Bajwa R. P. S. Russo T. A. (2013). Hypervirulent (hypermucoviscous) *Klebsiella pneumoniae*: a new and dangerous breed. *Virulence* 4 107–118. 10.4161/viru.22718 23302790 PMC3654609

[B68] Sims N. Kasprzyk-Hordern B. (2020). Future perspectives of wastewater-based epidemiology: monitoring infectious disease spread and resistance to the community level. *Environ. Int.* 139:105689. 10.1016/j.envint.2020.105689 32283358 PMC7128895

[B69] Snow J. (1855). *On the Mode of Communication of Cholera.* London: Macmillan.

[B70] Sodré F. F. Sampaio T. R. (2020). Development and application of a SPE-LC-QTOF method for the quantification of micropollutants of emerging concern in drinking waters from the Brazilian capital. *Emerg. Contaminants* 6 72–81. 10.1016/j.emcon.2020.01.001

[B71] Starikova E. V. Tikhonova P. O. Prianichnikov N. A. Zdobnov E. M. Govorun V. M. (2020). Phigaro: high throughput prophage sequence annotation. *Bioinformatics* 36, 3882–3884. 10.1093/bioinformatics/btaa250 32311023

[B72] Stoesser N. Sheppard A. E. Peirano G. Anson L. W. Pankhurst L. Sebra R. (2017). Genomic epidemiology of global *Klebsiella pneumoniae* carbapenemase (KPC)-producing *Escherichia coli*. *Sci. Rep.* 7:5917. 28725045 10.1038/s41598-017-06256-2 PMC5517641

[B73] Storey D. McNally A. Åstrand M. sa-Pessoa Graca Santos J. Rodriguez-Escudero I. Elmore B. (2020). *Klebsiella pneumoniae* type VI secretion system-mediated microbial competition is PhoPQ controlled and reactive oxygen species dependent. *PLoS Pathog.* 16:e1007969. 10.1371/journal.ppat.1007969 32191774 PMC7108748

[B74] Swick M. C. Morgan-Linnell S. K. Carlson K. M. Zechiedrich L. (2011). Expression of multidrug efflux pump genes *acrAB-tolC*, *mdfA*, and *norE* in *Escherichia coli* clinical isolates as a function of fluoroquinolone and multidrug resistance. *Antimicrob. Agents Chemother.* 55 921–924. 10.1128/AAC.00996-10 21098250 PMC3028778

[B75] Tram V. O. P. Ngo H. H. Guo W. Zhou J. L. Nguyen P. D. Listowski A. (2014). A mini-review on the impacts of climate change on wastewater reclamation and reuse. *Sci. Tot. Environ.* 494–495 9–17. 10.1016/j.scitotenv.2014.06.090 25020098

[B76] Treangen T. J. Ondov B. D. Koren S. Phillippy A. M. (2014). The Harvest suite for rapid core-genome alignment and visualization of thousands of intraspecific microbial genomes. *Genome Biol.* 15:524. 10.1186/s13059-014-0524-x 25410596 PMC4262987

[B77] Wick R. R. Heinz E. Holt K. E. Wyres K. L. (2018). Kaptive web: user-friendly capsule and lipopolysaccharide serotype prediction for *Klebsiella* genomes. Diekema DJ, organizador. *J. Clin. Microbiol.* 56 e197–e118. 10.1128/JCM.00197-18 29618504 PMC5971559

[B78] World Health Organization [WHO] (2015). *Global Action Plan on Antimicrobial Resistance [Internet]*. Disponível Em. Available online at: www.who.int/iris/handle/10665/193736 (accessed April, 2021).

[B79] Wozniak A. Figueroa C. Moya-Flores F. Guggiana P. Castillo C. Rivas L. (2020). A multispecies outbreak of carbapenem-resistant bacteria harboring the blaKPC gene in a non-classical transposon element. *BMC Microbiol.* 21:107. 10.1186/s12866-021-02169-3 33836654 PMC8034096

[B80] Wyres K. L. Holt K. E. (2018). *Klebsiella pneumoniae* as a key trafficker of drug resistance genes from environmental to clinically important bacteria. *Curr. Opin. Microbiol.* 45 131–139. 10.1016/j.mib.2018.04.004 29723841

[B81] Wyres K. L. Lam M. M. C. Holt K. E. (2020). Population genomics of *Klebsiella pneumoniae*. *Nat Rev Microbiol.* 18 344–359. 32055025 10.1038/s41579-019-0315-1

[B82] Yang X. Dong N. Chan E. W.-C. Zhang R. Chen S. (2021). Carbapenem resistance-encoding and virulence-encoding conjugative plasmids in *Klebsiella pneumoniae*. *Trends Microbiol.* 29 65–83. 10.1016/j.tim.2020.04.012 32448764

[B83] Zhang X. Li F. Cui S. Mao L. Li X. Awan F. (2020). Prevalence and distribution characteristics of bla_*KPC–2*_ and bla_*NDM–1*_ genes in *Klebsiella pneumoniae*. *Infect. Drug Resist.* 13 2901–2910. 10.2147/idr.s253631 32903853 PMC7445519

[B84] Zhou Z. Alikhan N.-F. Sergeant M. J. Luhmann N. Vaz C. Francisco A. P. (2018). GrapeTree: visualization of core genomic relationships among 100,000 bacterial pathogens. *Genome Res.* 28 1395–1404. 10.1101/gr.232397.117 30049790 PMC6120633

