# Supplementary figures and images for: A Novel Multidrug Resistant, Non-Tn4401 Genetic Element-Bearing, Strain of Klebsiella pneumoniae Isolated From an Urban Lake With Drinking and Recreational Water Reuse
Source: Front Microbiol. 2021 Nov 24;12:732324. doi: 10.3389/fmicb.2021.732324 (PMC8654192; doi:10.3389/fmicb.2021.732324)

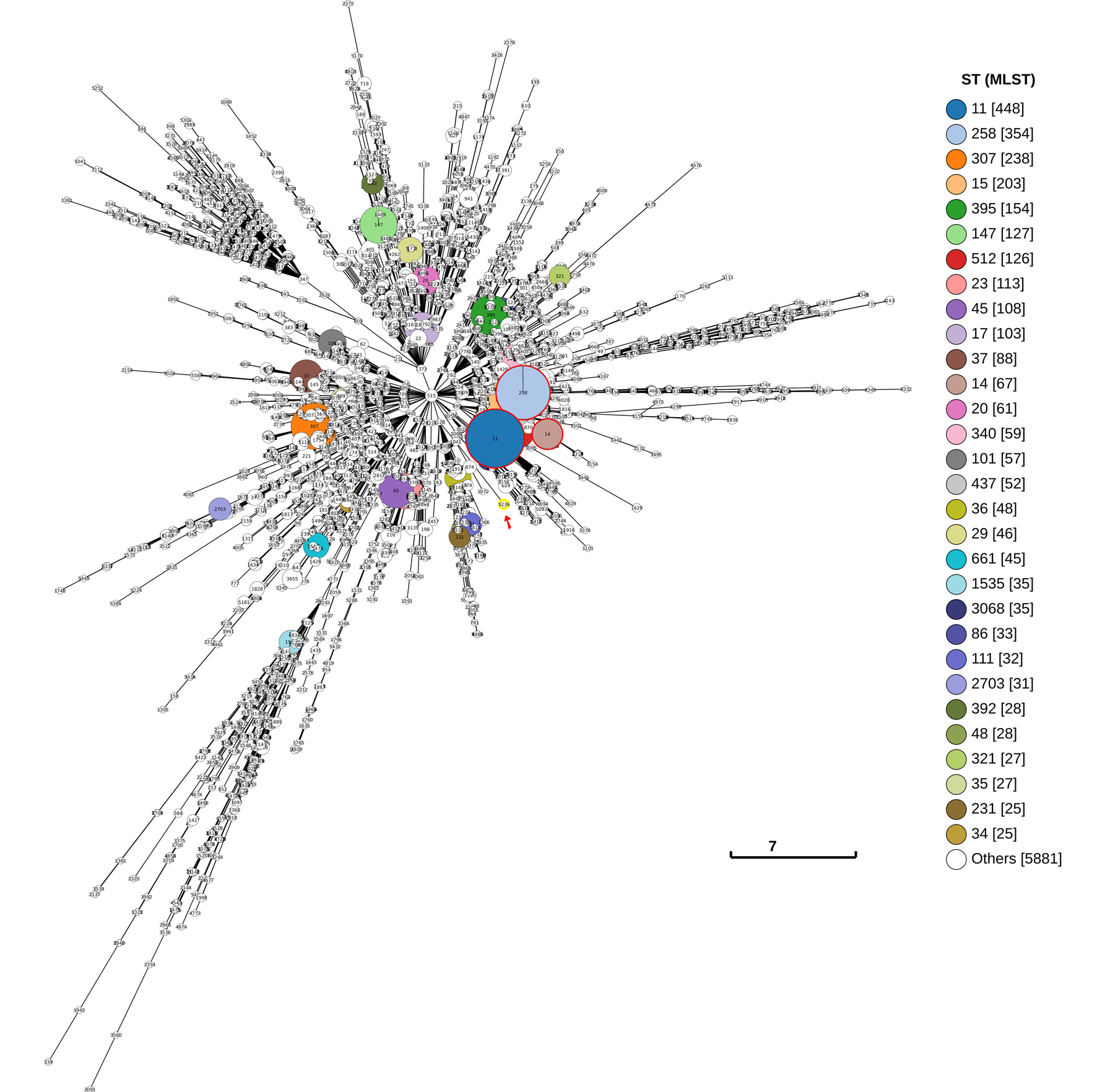

Supplement: Supplementary Figure 1 — Tree representation of genetic relationships among the different profiles of the K. pneumoniae MLST scheme. This analysis was produced via GrapeTree with the minimum spanning tree algorithm (MSTree V2). The ST identified in this study is highlighted with a yellow circle and a red arrow. High-risk lineages (CG 11, 14, and 258) are highlighted with red circles. [file Image_1.TIFF]
