# Supplementary material for: A Novel Multidrug Resistant, Non-Tn4401 Genetic Element-Bearing, Strain of Klebsiella pneumoniae Isolated From an Urban Lake With Drinking and Recreational Water Reuse
Source: Front Microbiol. 2021 Nov 24;12:732324. doi: 10.3389/fmicb.2021.732324 (PMC8654192; doi:10.3389/fmicb.2021.732324)
Supplement: Supplementary file 2 [file Table_1.DOCX]

Supplementary Table 1. Antibiogram results of KpV3

| Antibiotic | Halo diameter (mm) | Result |
| --- | --- | --- |
| Amikacin (30 µg) | 25 | Susceptible |
| Gentamicin (10 µg) | 20 | Susceptible |
| Tobramycin (10 µg) | 21 | Susceptible |
| Aztreonam (30 µg) | 0 | Resistant |
| Cefepime (30 µg) | 10 | Resistant |
| Imipenem (10 µg) | 19 | Resistant |
| Meropenem (10 µg) | 17 | Resistant |
| Piperacillin/tazobactam (100/10 µg) | 9 | Resistant |
| Ticarcillin/Clavulonate (85 µg) | 0 | Resistant |
| Ofloxacin (5 µg) | 0 | Resistant |
| Norfloxacin (10 µg) | 20 | Resistant |
| Ciprofloxacin (5 µg) | 20 | Resistant |
| Levofloxacin (5 µg) | 0 | Resistant |
| Lomefloxacin (10 µg) | 0 | Resistant |
